# Grafts of the Dental Follicles

**Published:** 1877-11

**Authors:** M. M. Ch. Segros, E. Magitot


### SELECTIONS.

### GRAFTS OF THE DENTAL FOLLICLES AND THEIR CONSTITUENT ORGANS. SEPARATELY.

BY M. M. CH. SEGROS AND E. MAGITOT.

[*Translated from the French for the Dental Register, by M. S. Dean.*]

We have the honor of bringing to the knowledge of *l'Academie*, the results which we have obtained in our efforts at animal grafting.

In these experiments we have not followed in the wake of most experimenters who have, since Hunter and the Italians, transplanted adult organs or tissues, but following out the idea already indicated by M. Bert, we have undertaken to engraft some embryonal organs or tissues; and among these, the dental follicle and the organs constituting it, which have, to us, appeared exceedingly favorable for that purpose.

Our experiments comprise eighty-eight grafts, divided into fourteen series. We can give here only a brief summary of them, followed by the results they have furnished, and some conclusions that may be drawn therefrom.

All the grafts have been taken from puppies, either new-born or a few days old. In a few experiments the puppies were from twenty-two to fifty-eight days old. The animals were invariably sacrificed and the jaws directly opened in order to lay bare the follicles. One-half of the two jaws furnished the grafts, while the other was preserved for future comparison.

The animals in which the grafts were inserted were generally adults, though sometimes of the same age, and of the same litter as those from which the grafts were taken. The organs experimented with were quickly taken from the dental grooves and most generally introduced directly into the part selected for the graft. In some cases they were left several minutes in the serum of the blood of the animal sacrificed, the serum being kept, by the wet bath, at a temperature of thirty to thirty-five degrees centigrade.

The grafts were introduced under the skin, in parts least liable to be disturbed by the movements of the animal. The nape of the neck, the top of the head, and in the cervical, dorsal, and lumbar regions. In the first series of experiment the process was to introduce the graft into a simple incision from two to three centimeters beneath the opening, which was re-united by a couple of stitches of a suture. This course was adopted in thirty-six of our experiments. In the fifty-two others we used a trocar, especially adapted to this purpose, of an interior diamater of seven millimeters, and which made the transplantation much more rapid and more sure. We ought to say, however, that the last method did not appear to us to exercise any appreciable influence on the results.

Of our total number of eighty-eight grafts, ten were from new-born puppies inserted in some adult guinea-pigs. They were divided in the following manner: Entire follicles, 6; enamel organs, separately, 3; the bulb* alone, 1. All these experiments ended in negative results, by resorption or suppuration, and sustain the facts gathered by M. Bert and other physiologists, in respect to grafts upon animals of different zoological orders.

The seventy-eight other grafts were invariably taken from new-born or young puppies, and engrafted into adults. The time during which these grafts were kept before examination, varied from thirteen to fifty-four days. We will say, in this connection, that of a series of twenty-five grafts, which had thus remained the maximum time of fifty-four days, all were wholly resorbed.

* The bulb is the dentine germ, or the part of the follicle which is destined to become dentine, and is often called dentine papilla.—*Tr.*

These seventy-eight grafts were divided in the following manner :

| | |
|---|---|
| A portion of the jaws, including the follicle | 5 |
| Entire follicles, isolated | 26 |
| Dental bulbs, isolated | 16 |
| Bulbs covered with a rudimentary dentine cap | 7 |
| Enamel organs isolated, engrafted with a shred of the mucous membrane. | 19 |
| Enamel organs with the cap of dentine | 1 |
| Caps of dentine, isolated | 4 |
| Total | 78 |

Of the five portions of the jaws, including the dental follicle, three resulted in suppuration, and two in resorption. M. Bert, in some similar attempts, met with the same negative results.

The nineteen grafts of the enamel organ isolated, and also those with the subjacent dentine cap, invariably terminated by resorption. This result is not surprising, when we consider the extreme fragility of this tissue and its absence from vascularity. We have, however, been careful to engraft, at the same time, the shred corresponding with the mucous membrane, which furnishes to the enamel organ its nutritive vessels.

Of the twenty-six complete follicles transplanted, seven of them continued to live, making a proportion of nearly twenty-five per cent. These seven follicles grew regularly, excepting in one case, in which some nutritive disturbance led to the production of globular dentine, and to fasciular irregularities of the prisms of the enamel.

The sixteen grafts of the dental bulbs alone gave three positive results, about twenty per cent of the number grafted. A new cap of dentine was reproduced, altogether regular in two cases, and in the third a little impaired in its nutrition and globular. They were wholly destitute of enamel.

The seven grafts of dental bulbs, covered with their caps of dentine, could not be found. They had been resorbed. This result is of surprising nature when compared with the preceding cases, but we will remark that these seven grafts belong to the series in which the experiments were continued during forty-three and fifty-four days.

Of the four isolated dentine caps, only one lived, but without growth.' It was found after the forty-third day in a stationary state.

A certain number of other grafts recorded in our statistics as negative, have been found either reduced in volume or manifestly in progress of resorption, or having undergone fatty degeneration, —a fact noticed several times by M. Bert; the others resulted in abscess and were eliminated, and others still could not be found, even by the most careful research.

Conclusions.—From the whole number of the preceding experiments, it seems possible to draw the following conclusions:

1. The grafts of dental follicles, or of follicular organs isolated, have not given successful results in our experiments, only between animals of the same zoological order.

2. The experiments in transplanting portions, (more or less voluminous, of the jaws), including the follicles, have failed through suppuration or resorption.

3. The grafts of the enamel organ separately appear to be destined invariably to resorption.

4. The entire follicles, and the dental bulb isolated, may continue to live and to devolop.

5. Under certain circumstances, the growth is accomplished regularly without differing from the normal state, except in a notable slowness in the phenomena of evolution.

6. In other circumstances, some defects in the formation of the dentine and the enamel are produced, and their study may be usefully applied to the research of phenomena still so obscure in the development of the dental organ.

7. The results, which flow from these experiments, can thus be united to those which are already acquired, in the line of surgical grafts.

